# *Z*-scheme 2D-m-BiVO_4_ networks decorated by a g-CN nanosheet heterostructured photocatalyst with an excellent response to visible light†

**DOI:** 10.1039/c9ra09473c

**Published:** 2020-01-20

**Authors:** Toheed Ahmed, Muhammad Ammar, Aimen Saleem, Hong-ling Zhang, Hong-bin Xu

**Affiliations:** Department of Applied Chemistry, Government College University Faisalabad 38000 Pakistan toheedahmed@gcuf.edu.pk; Key Laboratory of Green Process and Engineering, Institute of Process Engineering, Chinese Academy of Sciences Beijing 100190 China; University of Chinese Academy of Sciences Beijing 100049 China; Department of Chemical Engineering Technology, Government College University Faisalabad 38000 Pakistan mammar@gcuf.edu.pk +92419203027 +92419203027; Biotechnology and Fermentation Group, Department of Animal Sciences, The Ohio State University, QARDC Wooster Ohio 44691 USA

## Abstract

For economical water splitting and degradation of toxic organic dyes, the development of inexpensive, efficient, and stable photocatalysts capable of harvesting visible light is essential. In this study, we designed a model system by grafting graphitic carbon nitride (g-C_3_N_4_) (g-CN) nanosheets on the surface of 2D monoclinic bismuth vanadate (m-BiVO_4_) nanoplates by a simple hydrothermal method. This as-synthesized photocatalyst has well-dispersed g-CN nanosheets on the surface of the nanoplates of m-BiVO_4_, thus forming a heterojunction with a high specific surface area. The degradation rate for bromophenol blue (BPB) shown by BiVO_4_/g-CN is 96% and that for methylene blue (MB) is 98% within 1 h and 25 min, respectively. The 2D BiVO_4_/g-CN heterostructure system also shows outstanding durability and retains up to ∼95% degradation efficiency for the MB dye even after eight consecutive cycles; the degradation efficiency for BPB does not change too much after eight consecutive cycles as well. The enhanced photocatalytic activities of BiVO_4_/g-CN are attributed to the larger surface area, larger number of surface active sites, fast charge transfer and improved separation of photogenerated charge carriers. We proposed a mechanism for the improved photocatalytic performance of the *Z*-scheme photocatalytic system. The present work gives a good example for the development of a novel *Z*-scheme heterojunction with good stability and high photocatalytic activity for toxic organic dye degradation and water splitting applications.

## Abstract 2

For economical water splitting and degradation of toxic organic dyes, the development of inexpensive, efficient, and stable photocatalysts capable of harvesting visible light is essential.

## Graphical abstract

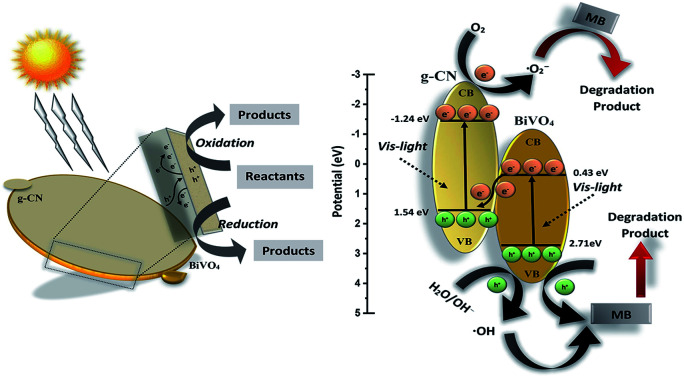

## 1 Introduction

Due to the environmental and growing energy crisis, more attention has been paid to the exploration of highly efficient photocatalyst materials that can endorse the direct use of abundant solar energy resources to drive several reactions.^1^ Heterostructures from two-dimensional (2D) materials offer a particularly new stage for exploring new physics. Prominently, 2D heterojunctions can facilitate the separation of photoexcited electrons and holes.^2^ Recently, an organic 2D nonmetallic semiconductor planar structure with a π conjugated system, namely, g-CN with a band gap of ∼2.7 eV has been discovered as a promising candidate in the field of photodegradation and photocatalytic reactions such as water splitting, CO_2_ conversion, and polluted water treatment for its thermal stability, easy availability and suitable band structure/location.^3–6^ Unfortunately, pristine g-CN still has some shortcomings, such as massive photo-induced charge carrier recombination and moderate bandgap.^7^ For the sake of enhanced photocatalytic efficiency of g-CN, copious strategies have been exploited, such as metal deposition,^8,9^ texture engineering^10,11^ and doping.^3,12^ Among these various methods, coupling g-CN with another semiconductor with a narrow band gap will enhance the solar spectrum response, enable considerable alternations of the electronic band structure and efficiently suppress the recombination of photo-induced electron–hole pairs by charge transfer; thus, it is a dominant research topic in the recent decade and is going to be a pivotal one to increase the efficiency of photocatalysts.

Among the photo-driven semiconductors, m-BiVO_4_ has attracted more attention due to nontoxicity, relatively high photocatalytic activity and chemical stability for the degradation of organic compounds and water splitting,^13–15^ but BiVO_4_ alone cannot degrade dyes and split water efficiently because its conduction band is located at a more positive potential than the potential of water reduction [0 eV *vs.* NHE; H^+^/H_2_].

In order to solve this problem, many strategies such as doping with nonmetallic elements,^16^ combined with graphene^17^ and constructing heterojunctions with two semiconductors, have been employed.^18^ Among the variety of photocatalytic systems, the direct solid-state *Z*-scheme system has attracted considerable attention because it not only enhances the spatial separation efficiency of photo-induced electron–hole pairs but also reduces the undesirable backward reaction of the photocatalytic process due to two different redox sites.^19,20^ Furthermore, maximum overpotentials can be achieved with this unique *Z*-scheme system, which is beneficial for the effective utilization of a high conduction band from a semiconductor and a low valence band from another semiconductor.^21–23^ Li *et al.* synthesized a *Z*-scheme BiVO_4_/g-CN photocatalytic system by thermal annealing and hydrothermal method.^24^ The development of a reliable and efficient methodology to obtain reusable and stable heterostructure photocatalysts with optimized activity is still a great challenge. Based on the afore-mentioned results, controllable surface coverage is significant in the *Z*-scheme system to achieve highly efficient photocatalysts. So far, there are few reports on the construction of m-BiVO_4_-based *Z*-scheme ternary heterostructures for the photodegradation of bromophenol blue (BPB).

Herein, we demonstrate the construction of an efficient 2D heterojunction BiVO_4_/g-CN photocatalyst model system with 2D-m-BiVO_4_ nanoplates covered by discrete g-CN nanosheets with controllable surface coverage. To construct the heterojunction photocatalyst, g-CN and m-BiVO_4_ were selected with the following considerations: primarily, both g-CN and BiVO_4_ have been proven to be promising visible-light photocatalysts with desirable chemical stability. Second, the proper energy-band alignments at the heterojunction interface are crucial and beneficial for the light-induced separation of charge carriers in the as-synthesized heterojunction photocatalyst system. Predominantly, the heterojunction structures of BiVO_4_/g-CN can be simply modified to attain a controllable coverage of g-CN on the surface of BiVO_4_ by the hydrothermal process. These features provide us with a good platform to get insights into the significance of heterostructure engineering for fabricating heterojunction photocatalysts.

## 2 Experimental section

### 2.1. Materials

**Scheme 1:**
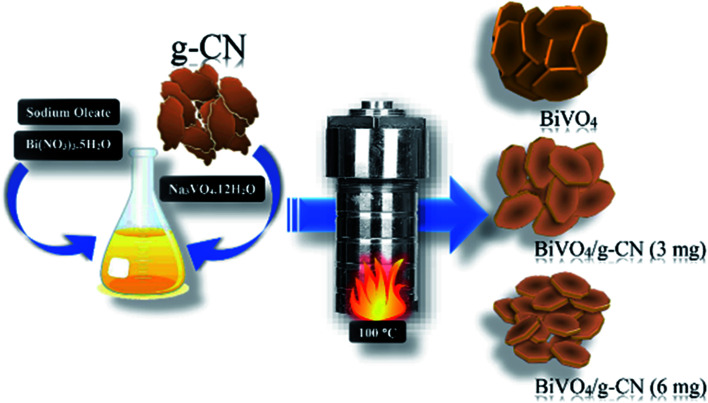
Schematic illustration of the BiVO_4_/g-CN heterostructure photocatalyst system.

All chemicals were of analytical grade, obtained from commercial sources and were used without further purification. The preparation of g-CN nanosheets was conducted according to a previous report by following the combustion technique with some modifications. Ten grams of urea was placed in a silica crucible and then calcined at 550 °C for 2 h.^25^ A certain amount of bulk g-CN was dispersed in H_2_SO_4_ and stirred for 7 h; then, it was washed with deionized water and dried at 80 °C to obtain the g-CN nanosheets. The m-BiVO_4_/g-CN heterojunction photocatalyst was prepared by the hydrothermal method, as illustrated in Scheme 1. First, sodium oleate (1.5 mmol) and Bi(NO_3_)_3_·5H_2_O (0.4 mmol) were put into 20 mL distilled water. Then, Na_3_VO_4_·12H_2_O (0.4 mmol) was dissolved in 20 mL distilled water, followed by sonication until a transparent solution was obtained; then, a certain amount of g-CN nanosheets were added in it and the mixture was placed on a magnetic stirrer for 1 h. The obtained solutions in the first and second steps were named as A and B, respectively. Solution A was mixed dropwise into solution B at the rate of 100 drops per minute under vigorous stirring for 2 h. Progressively, the homogeneous yellow color solution was transferred into a Teflon-lined stainless-steel autoclave (50 mL), sealed and maintained at 100 °C temperature for 12 h and then cooled down to room temperature. Finally, the obtained product was separated by centrifugation and washed successively with distilled water and ethanol. Then, the final product was dried under a vacuum furnace at 60 °C for 6 h. The obtained samples were named according to the amount of g-CN as m-BiVO_4_/g-CN (weight of g-CN).

### 2.2. Characterization

The samples were characterized using different techniques. X-ray diffraction (PAN analytical Empyrean CuKα = 0.15406 nm, 40 kV, 40 mA) from 5° to 90° was used for phase and crystal structure studies. The morphology of samples was identified by scanning electron microscopy (SEM, Hitachi, Ltd., S4700). Reflectance spectroscopy was analyzed by a UV-3600 spectrophotometer (Shimadzu Corporation) in the wavelength range of 200–800 nm with barium sulphate as the reference. X-ray photoelectron spectroscopy (XPS) characterization was carried out on an ESCALAB 250Xi (Thermo Fisher Scientific Inc.) spectrometer using an Al Kα source. Specific surface areas were measured by Brunauer–Emmett–Teller (BET) nitrogen adsorption–desorption at 77 K on Micromeritics ASAP-2010. The electrochemical workstation CH1760E equipped with a Xe lamp (AM 1.5G) was used for photoelectron measurements. Electron spin-resonance spectroscopy (ESR) was conducted on a Bruker model EMX 10/12 spectrometer (Bruker, Germany) equipped with a Hg lamp for the measurement of the signals of radicals spin-trapped by 5,5-dimethyl-1-pyrroline *N*-oxide (DMPO) at a microwave frequency of 9.77 GHz.

### 2.3. Evaluation of photocatalytic activity

A homemade device was used for the photocatalytic degradation test, which was equipped with a 500 W xenon arc lamp to simulate sunlight. For the photocatalytic experiment, 0.1 g of photocatalyst sample was put into a glass tube containing 100 mL aqueous solution of methylene blue (MB) (5 mg L^−1^) or BPB (5 mg L^−1^). Immediately the glass tube was placed into the photocatalytic degradation test box under darkness. Then, the solution was stirred continuously for 1 h to achieve adsorption equilibrium. After 1 h, the photocatalyst–MB dye suspension was continuously stirred under stimulated sunlight irradiation. The distance between the upper surface of the suspension and the lamp was 16 cm. A UV-vis spectrophotometer (Hach USA, DR-5000) was used to measure the photocatalytic discoloration rate of MB with and without a sample.

### 2.4. Fabrication of electrodes for photoelectrochemical measurements

For electrode preparation, 5 mg of g-CN/m-BiVO_4_ photocatalyst powder was dispersed in an equal ratio of 200 μL ethanol and deionized water. This solution was ultrasonicated for about 8 hours and 30 μL Nafion as the chemical binder was added into the prepared photocatalyst solution and sonicated further for 3 h to get a homogeneous dispersion. This slurry was then coated on a washed piece of FTO as a transparent conducting substrate and annealed in air overnight at 60 °C to dry it and to increase the adhesion between the substrate and the coated film.

### 2.5. Photoelectrochemical water oxidation measurements

A photoelectrochemical analyzer (Zahner Zennium, Germany) was used to test the photoelectrochemical properties of the samples in a conventional three-electrode cell. The working electrode was made by coating the photocatalyst on FTO. A Pt sheet was used as the counter electrode and a saturated Ag/AgCl (SCE) electrode was used as the reference electrode. The working electrode was irradiated by a Xe lamp (CEL-S500, Aulight, Beijing: 500 W, luminous power: 50 W) and the light density was calibrated to be 100 mW cm^−2^ (1 Sun). Also, 1 M Na_2_SO_4_ aqueous solution was used as the supporting electrolyte. The working electrode was prepared as follows: 5 mg of the photocatalyst sample was suspended in 600 μL ethanol and sonicated for 4 h, followed by the addition of Nafion (5 wt%). Then, 100 μL of the slurry was drop-casted onto FTO with a working area of 1 cm^2^. The cathodic polarization curves were obtained using the linear sweep voltammetry (LSV) technique with a scan rate of 0.5 mV s^−1^. Electrochemical impedance spectroscopy (EIS) was performed under simulated AM 1.5G sunlight on an Autolab workstation (AUT85812) equipped with a frequency analyzer module based on the above three-electrode system in 0.2 M Na_2_SO_4_.

### 2.6. Analysis of hydroxyl radicals

The formation of the hydroxyl radicals (OH˙) on the surface of the photocatalyst under visible light irradiation was detected by the photoluminescence (PL) method using terephthalic acid as a probe molecule. Terephthalic acid reacts with hydroxyl radicals to form hydroxylterephthalic acid, which is highly fluorescent.^26,27^ The method depends on the PL signals at around 425 nm arising from the hydroxylation of terephthalic acid with hydroxyl radicals generated at the water–catalyst interface. The fluorescence intensity of 2-hydroxyterephthalic acid is proportional to the number of hydroxyl radicals produced in water. The method is sensitive, rapid and specific. The detection experiment was similar to the measurement of photocatalytic degradation activity (2.3) except that the MB medium was replaced by 5 × 10^−4^ mol L^−1^ terephthalic acid in dilute NaOH (2 × 10^−3^ mol L^−1^) solution. The photocatalyst (200 mg) was added to 50 mL solution, which was kept in the dark for 30 min before irradiation to ensure desorption/adsorption equilibrium. A 500 W xenon lamp was used (luminous flux 5–15 lm W^−1^, ultraviolet-radiation kept below 242 nm). The sample was taken out every 10 min and after centrifugation, the PL spectra of the generated 2-hydroxyterephthalic acid were measured on Tecan infinite M200 after being excited by the wavelength of 315 nm.

### 2.7. Active species trapping

The active species during the photocatalytic reaction were determined by using some sacrificial agents, such as 1,4-benzoquinone (BQ), ammonium oxalate (AO) and *tert*-butanol (*t*-BuOH), which could capture superoxide radicals (O_2_˙^−^), holes (h^+^) and hydroxyl radicals (OH˙), respectively. The method was similar to the earlier photocatalytic activity test (2.3) with the addition of 1 mmol quencher in the presence of MB.

## 3 Results and discussion

### 3.1. Physiochemical property analysis

**Fig. 1:**
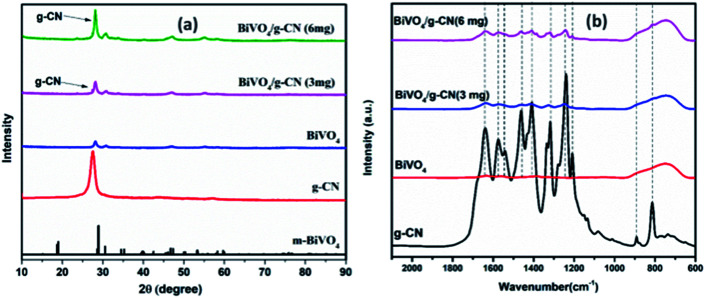
(a) XRD patterns of the as-prepared samples. (b) FTIR spectra of the prepared samples.

The crystalline structure of the prepared samples was investigated by XRD analysis. Fig. 1(a) presents the XRD analysis of pure g-CN, BiVO_4_ and BiVO_4_/g-CN. As shown in Fig. 1(a), the BiVO_4_/g-CN composites with various amounts of g-CN exhibit similar diffraction patterns to that of individual BiVO_4_. The calculated cell parameters of pure BiVO_4_ were *a* = 0.5106 ± 0.0016 nm, *b* = 1.1720 ± 0.0026 nm, and *c* = 0.511 ± 0.0010 nm (JCPDS card no. 83-1699). The XRD peak at 27.5° corresponds to the inter-planar stacking of the conjugated aromatic systems of g-CN.^28^Fig. 1(a) shows that the characteristic diffraction peak of g-CN in the BiVO_4_/g-CN composite slightly shifts towards a higher value (∼27.9°), which may be due to the decreased interplanar spacing between the g-CN and BiVO_4_ sheets.^29^ Specifically, pure g-CN shows a relatively broad diffraction peak due to its amorphous nature, while the BiVO_4_/g-CN composite exhibits semi-crystalline behavior.

In order to investigate the surface chemistry and the presence of various groups in the prepared samples, the relative FTIR spectra are displayed in Fig. 1(b). For pure BiVO_4_, a peak at 740 cm^−1^ can be attributed to the *ν*_3_ asymmetric stretching vibration of the VO_4_ unit, while the other peak at 843 cm^−1^ can be identified as the *ν*_1_ symmetric stretching vibration of the VO_4_ unit.^30^ For pure g-CN, the typical FTIR spectrum of g-CN can be observed. A series of peaks in the region of 1200–1800 cm^−1^ belong to the typical stretching modes of heterocyclic CN. For example, the peak locates at 807 cm^−1^ corresponds to the out-of-plane bending vibration of the g-CN triazine unit.^31,32^ The FTIR spectra of all the BiVO_4_/g-CN nanocomposites contain almost all characteristic peaks of BiVO_4_ and g-CN, indicating the co-existence of both structures. By increasing the amount of g-CN, the peak intensities of g-CN increased for the BiVO_4_/g-CN nanocomposite samples.

**Fig. 2:**
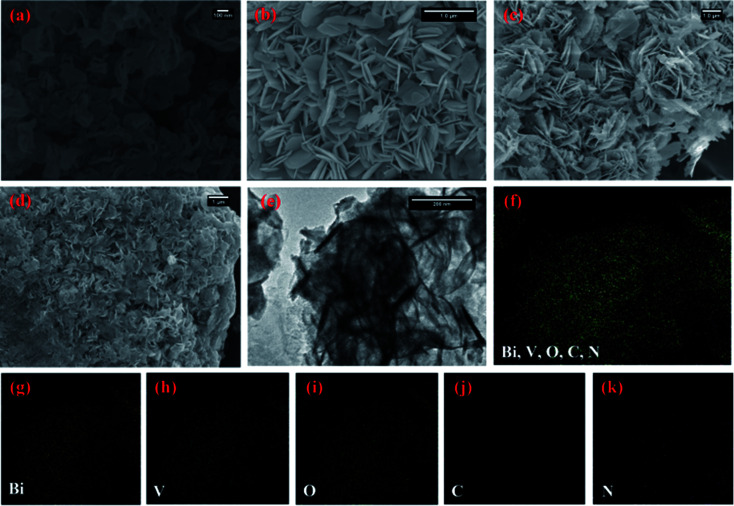
SEM images of (a) bulk g-CN and (b) BiVO_4_ prepared at 100 °C, (c) BiVO_4_/g-CN (3 mg) and (d) BiVO_4_/g-CN (6 mg); (e) HRTEM image of BiVO_4_/g-CN (6 mg). (f–k) Elemental mapping of BiVO_4_/g-CN (6 mg).

Fig. 2(a) depicts the morphology of the g-CN nanosheets having ∼5 nm thicknesses. The pristine BiVO_4_ sample exhibits homogeneous nanoplate morphology (Fig. 2(b)). The average diameter of the nanoplates was ∼100 nm with an average thickness of ∼15 nm. With the increase in the initial amount of g-CN from 3 to 6 mg in the BiVO_4_/g-CN composite, the nanoplates became thinner and smaller in diameter. Numerous nanosheets can be clearly seen covering the BiVO_4_ nanoplates, which may be due to g-CN (Fig. 2(c and d)). The particle size of BiVO_4_ is much smaller and the particles are distributed between the nanosheets of g-CN, inferring that the agglomeration of BiVO_4_ is well prevented by the nearby g-CN nanosheets. To confirm the phase distribution and the network morphologies, a high-resolution transmission electron microscopy (HRTEM) image of the BiVO_4_/g-CN (6 mg) composites is shown in Fig. 2(e). It is apparent that the phase with a nanoplate shape shows a high degree of crystallinity for a pure lattice m-BiVO_4_ phase. Conversely, the g-CN nanosheets covering the BiVO_4_ networks showed low crystallinity with fuzzy lattices. It is noteworthy that the two phases are in contact with each other through a well-defined sharp interface boundary, which indicates the formation of a high-quality BiVO_4_/g-CN heterojunction structure. Specifically, g-CN formed a continuous sheet on the surface of BiVO_4_ with a thickness from 7 nm to 5 nm. Furthermore, the scanning transmission electron microscopy (STEM) elemental mapping confirmed the discrete dispersion of the g-CN phase on the BiVO_4_ surface (Fig. 2(g–k)).

Moreover, Fig. 2(f) and HRTEM image (Fig. S1 in ESI†) prove that the top surface of BiVO_4_ corresponds to the (121) facet. Fig. S1† displays that the nanosheets and nanoplates attached to each other, with the (002) facets of g-CN and the (121) facets of BiVO_4_, indicating that the coupling between g-CN and BiVO_4_ may happen on the (002) facets of g-CN and the (121) facets of BiVO_4_.

The BET surface areas of the pure BiVO_4_ and BiVO_4_/g-CN (6 mg) composite samples were investigated by nitrogen (N_2_) adsorption. The inset in Fig. S2† shows the N_2_ sorption isotherms and the corresponding distribution of pore size curves for the BiVO_4_/g-CN (6 mg) composite and pure BiVO_4_. The N_2_ adsorption–desorption isotherms for the afore-mentioned samples exhibit type-IV BDDT (Brunauer–Deming–Deming–Teller) classification, which indicates the existence of mesopores (widths of 24 and 32 nm). These isotherms showed H_3_ hysteresis loops related to the mesopores existing in aggregates composed of primary particles. In addition, the adsorption branch of the N_2_ isotherms firmly increased when *P*/*P*_0_ proceeded towards unity, indicating the formation of small macropores and large mesopores. Very broad pore size distributions of samples (inset in Fig. S2†) indicate the existence of both macropores and mesopores. The BiVO_4_/g-CN (6 mg) sample has a larger surface area than that of pure BiVO_4_ (Table S1†). The larger surface area of the composite is due to the existence of g-CN (∼31 m^2^ g^−1^) in the composite. It can be concluded that the specific surface area increase on increasing the amount of g-CN contributes in enhancing the photocatalytic performance of the composites.

The electronic interactions between BiVO_4_ and g-CN in the hybrid and the chemical states of the elements were studied by using X-ray photoelectron spectroscopy (XPS). Fig. S3(a and b)† demonstrate the survey spectra of pure BiVO_4_ and the BiVO_4_/g-CN (6 mg) composites, demonstrating the presence of all the elements of g-CN and BiVO_4_ in the composite material. The characteristic peak for N=C–N_2_ in g-CN is observed at 288.2 eV in the C 1s spectrum. The N 1s (Fig. S3(c)†) spectrum was deconvoluted into three typical peaks at 398.3, 399.2 and 400.7 eV due to the N 1s core level in the C–N=C bond, the bridging N atoms bonded to three C atoms (N–[C]_3_) and the N atoms in the C–N=H bonds, respectively. The C 1s peaks (Fig. S3(d)†) at 284.8 eV and 288.6 eV can be attributed to the adventitious carbon on the surface of g-CN.^33^ These results further confirmed that both BiVO_4_ and g-CN exist in the composite.

**Fig. 3:**
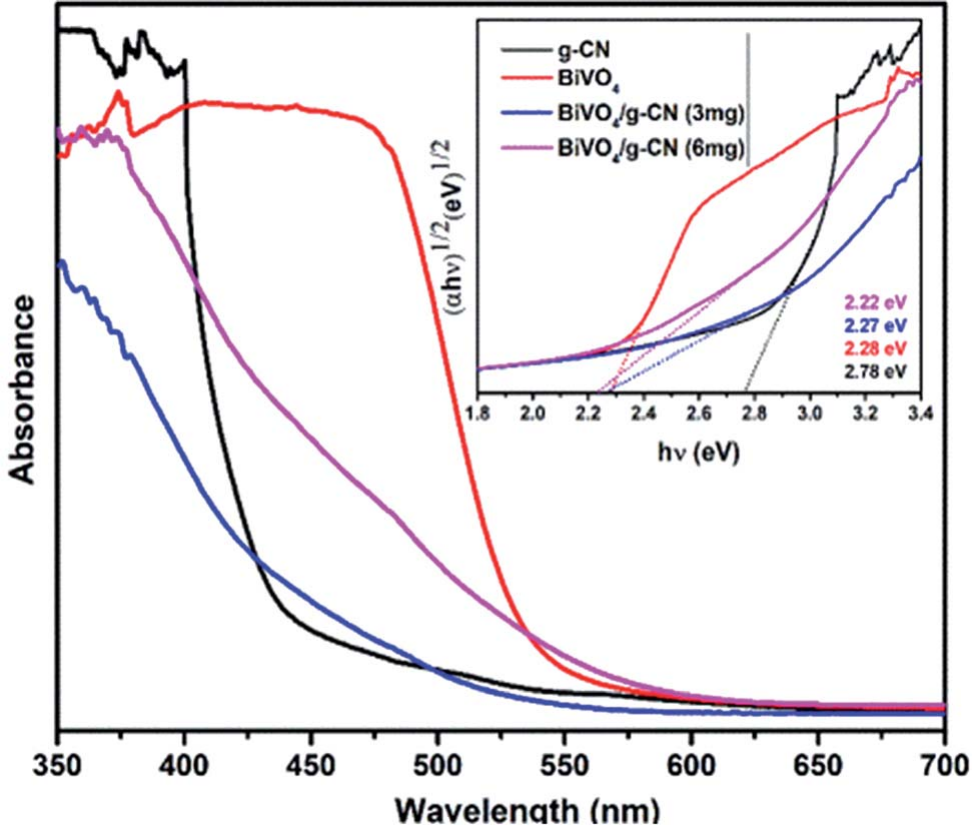
UV-visible spectra of the prepared samples and the corresponding plots of (*αhν*)^1/2^*versus* photon energy (*hν*).

Fig. 3 presents the diffuse reflectance spectra in the range of 400–700 nm for all samples. By increasing the amount of g-CN, the absorption of the BiVO_4_/g-CN samples in the visible light region probably the same. The steep shape of the curves indicated that the band gap transition was responsible for the visible light absorption. The optical absorption near the band edge is calculated using the equation *αhν* = *A*(*hν* − *E*_g_)^*n*^, where *A* is a constant, *α* is the absorption coefficient, *E*_g_ represents the band gap, *ν* is the light frequency and the value of *n* depends on the transition characteristics of the semiconductor (*n* = 2 for indirect transition and *n* = ½ for direct transition). For our system, m-BiVO_4_, the value of *n* is 2.^34^ The plot of photon energy (*αhν*)^1/2^*versus hν* for m-BiVO_4_ and the BiVO_4_/g-CN composite yields a straight line (inset in Fig. 3) and the intercept of the tangent to the *X*-axis used for the approximation of its band gap. As listed in Table 1, the approximate *E*_g_ values of m-BiVO_4_ and the BiVO_4_/g-CN composite vary from 2.22 eV to 2.28 eV. BiVO_4_/g-CN has a low *E*_g_ value and higher absorption, which is beneficial for enhancing its photocatalytic degradation performance. On the contrary, the band gap of pure g-CN exhibits a higher value of 2.78 eV, and it is not very efficient under visible light due to the high band gap value.

**Table 1:** Synthesis conditions for preparation of all samples and their physical properties

Sample	Temperature	Phase^a^	Surface area (m^2^ g^−1^)	Degradation (%) MB	Degradation (%) BPB	*k* (min^−1^) MB	*k* (min^−1^) BPB
BiVO_4_	100 °C	M	11	82	85	0.028	0.032
g-CN	100 °C	M	31	40	15	0.011	0.002
BiVO_4_/g-CN (3 mg)	100 °C	M	19	84	86	0.042	0.033
BiVO_4_/g-CN (6 mg)	100 °C	M	28	98	95	0.128	0.048

a M = monoclinic, *k* = rate constant.

### 3.2. Degradation of organic dye methylene blue

**Fig. 4:**
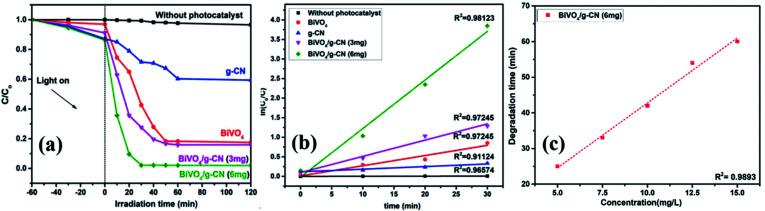
(a) Photocatalytic degradation of MB over samples under visible light irradiation. (b) The ln(*C*_0_/*C*) as a function of irradiation time (*t*) for MB degradation. (c) Degradation time of BiVO_4_ and BiVO_4_/g-CN (6 mg) as a function of dye concentration (5–15 mg L^−1^).

**Fig. 5:**
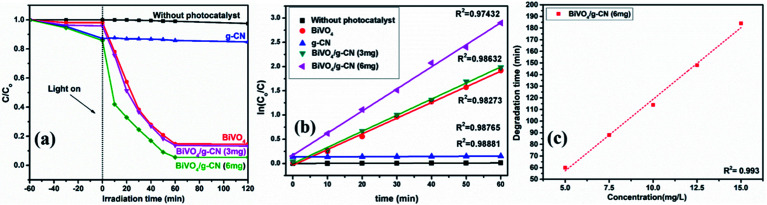
(a) Photocatalytic degradation of BPB over samples under visible light irradiation. (b) The ln(*C*_0_/*C*) as a function of irradiation time (*t*) for MB degradation. (c) Degradation time of BiVO_4_ and BiVO_4_/g-CN (6 mg) as a function of dye concentration (5–15 mg L^−1^).

The photoactivity of photocatalysts was evaluated by the degradation of the organic dyes MB and BPB under irradiation in the range of visible light (*λ* ≥ 420 nm), a common model to test the photodegradation efficiency.^35^ The degradation of MB and BMP over different photocatalyst samples is illustrated in Fig. 4(a) and 5(a), respectively. The results showed that the band gap and surface area might influence the photocatalytic degradation of MB and BMB. The BiVO_4_/g-CN photocatalysts exhibited higher photocatalytic activity than pure BiVO_4_ under visible-light irradiation for both dyes. The activity of BiVO_4_/g-CN (6 mg) was obviously superior to that of others; it showed 98% degradation of MB in 25 minutes and 95% degradation of BMB in 1 hour. It is also noted that g-CN shows very little degradation of MB and BMB under visible-light irradiation. The apparent result shows that the formation of the BiVO_4_/g-CN heterojunction composite structures greatly improve the photocatalytic degradation activities. Among all these photocatalysts, BiVO_4_/g-CN (6 mg) with discrete g-CN nano-sheets decorated on the BiVO_4_ networks showed the highest photocatalytic activity. The photocatalytic results confirm that the loading amount of g-CN plays a significant role in the photocatalytic performance of the BiVO_4_/g-CN composite.

In order to further depict the photocatalytic reaction, the photocatalytic degradation route of MB and BMB was fitted to a pseudo-first-order kinetics model ln(*C*_0_/*C*) = *kt* (Fig. 4(b) and 5(b)), where *k* is the reaction rate constant. The value of *k*, which is equal to the corresponding slope of the fitting line, was calculated and listed in Table 1. The value of *k* for an efficient photocatalyst is usually high. A sharp increase in *k* was observed on increasing the amount of g-CN present in the BiVO_4_/g-CN composite. When the amount of g-CN reached up to 6 mg in the BiVO_4_/g-CN composite, it exhibited the maximum photocatalytic activity. The rate constant of the BiVO_4_/g-CN (6 mg) composite was 0.128224 min^−1^ for MB, which was 4.5-fold higher than that of pure BiVO_4_; for BMB, the rate constant was 2.02 min^−1^, which was 1.5-fold higher than that of pure BiVO_4_. The interaction with the g-CN nanosheet layers formed in the presence of the BiVO_4_ nanoplates at the interface generated sufficient nano-junctions to hinder the re-stacking of the 2-D nanosheets. Therefore, the enlarged specific surface area of the as-obtained composite due to better surface coverage not only allowed access for the molecules of the dye to the surface but also promoted the transfer of surface carriers across the interfaces. Combined with the above-mentioned photo-electron chemical and optical analyses, it can be deduced that the efficiency enhancement in the photocatalytic degradation performance can be attributed to the better surface coverage of g-CN on the surface of BiVO_4_, improved charge separation by the well-matched band structure and enlarged specific surface area by the well contacted interface.

The pollutant concentration is a very important parameter in wastewater treatment. The effects of various initial dye concentrations (MB and BPB) on photocatalytic decolorization investigated from 5 to 15 mg L^−1^ against fixed amounts (0.1 g) of BiVO_4_ and BiVO_4_/g-CN (6 mg) were recorded and shown in Fig. 4(c) and 5(c), respectively. By the successive increment in the concentration, the degradation time increased linearly. However, superior degradation up to 96% could still be achieved in about 1 hour for up to 15 mg L^−1^ of MB by using the BiVO_4_/g-CN (6 mg) photocatalyst and for up to 15 mg L^−1^ of BPB, 94% degradation was achieved in about 3 hours using the same photocatalyst.

**Fig. 6:**
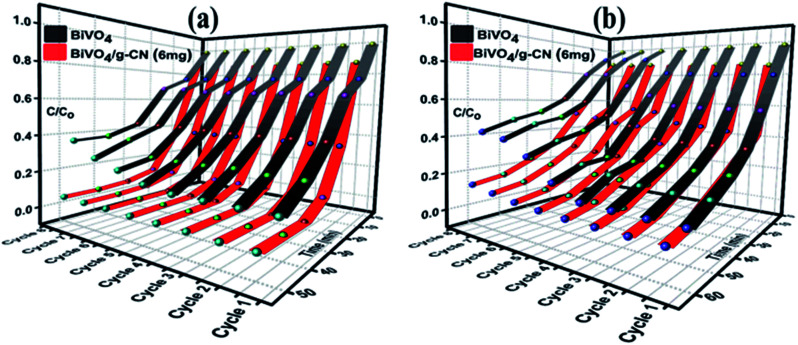
Recycle experiments of BiVO_4_ and BiVO_4_/g-CN (6 mg) nanocomposites for the photocatalytic degradation of (a) MB (b) BPB.

The reusability and stability of the BiVO_4_ and BiVO_4_/g-CN photocatalysts were assessed by recycling photodegradation experiments and the results for the degradation of MB and BMB over different photocatalyst samples are shown in Fig. 6(a and b). No significant loss in the photocatalytic activity of the BiVO_4_/g-C_3_N_4_ (6 mg) photocatalyst was observed after successive cycles; it showed 98% to 95% degradation activity after 8 cycles for MB and 95% to 86% degradation activity after 8 cycles for BMB, suggesting that the strong interfacial interaction between BiVO_4_ and g-CN benefits the stability of the heterostructure. Even after 8 successive photodegradation experiments, the BiVO_4_/g-C_3_N_4_ (6 mg) photocatalyst did not show any obvious decrease in the photocatalytic degradation activity under visible-light irradiation, implying that the BiVO_4_/g-CN photocatalyst is sufficiently stable for photocatalytic degradation. On the contrary, the efficiency of pure BiVO_4_ decreased after 5 successive runs against both dyes.

**Fig. 7:**
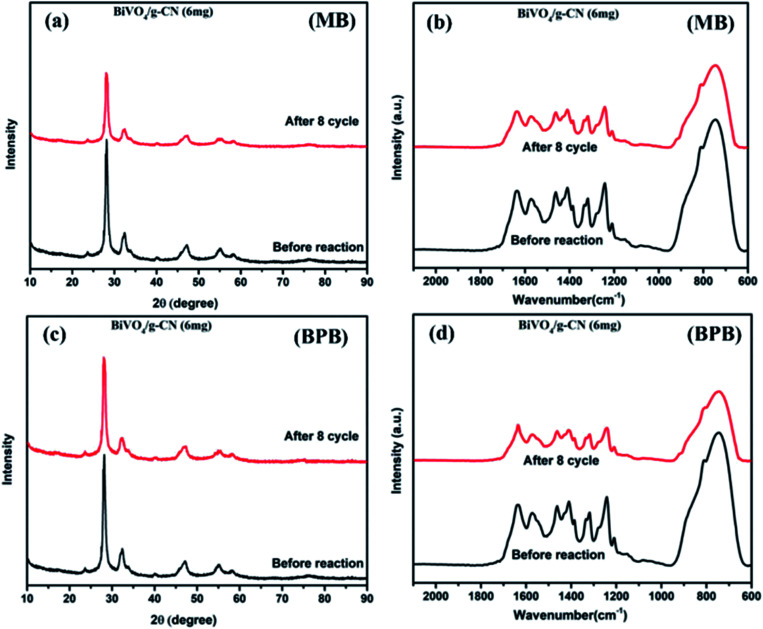
(a) The XRD pattern (MB), (b) FTIR pattern (MB) and (c) XRD pattern (BPB), (d) FTIR pattern (BPB) of BiVO_4_/g-CN (6 mg) before and after eight runs.

As further evidence, the XRD and FTIR patterns of the fresh and used hybrid photocatalyst are compared in Fig. 7 (a–d). All peak intensities decrease in the XRD spectra after 8 cycles, as shown in Fig. 7 (a and c). The FTIR spectra (Fig. 7 (b and d)) of BiVO_4_/g-CN (6 mg) show that the intensities at 740 cm^−1^, 843 cm^−1^ and 1200–1800 cm^−1^ decrease, which might be due to the increased surface strain of BiVO_4_/g-C_3_N_4_ caused by the adsorption of dye molecules. In contrast, no significant difference could be observed in absorption bands, which demonstrated the stable chemical structure during the whole reaction.

### 3.3. Water oxidation activity measurements

**Fig. 8:**
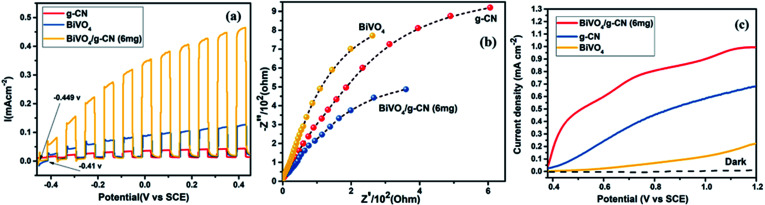
(a) Transient photocurrent responses of BiVO_4_, g-CN and BiVO_4_/g-CN (6 mg). (b) EIS Nyquist plots of BiVO_4_, g-CN and BiVO_4_/g-CN (6 mg). (c) The cyclic voltammograms of BiVO_4_, g-CN and BiVO_4_/g-CN (6 mg) in dark (dotted curves) and under illumination of simulated AM 1.5G sunlight.

The photoelectrochemical properties of the as-synthesized BiVO_4_/g-CN were evaluated by linear-sweep voltammetry under chopped light irradiation from a xenon lamp (AM 1.5G). Fig. 8(a) shows that the g-CN, m-BiVO_4_ and BiVO_4_/g-CN (6 mg) photocatalysts exhibit rapid rise in the photocurrent over a wide potential range when light is turned on and drop when the light is turned off, implying that photocurrents are generated under visible-light irradiation. The anodic current density of BiVO_4_/g-CN (6 mg) demonstrates the n-type semiconducting nature of the as-synthesized BiVO_4_/g-CN (6 mg) photocatalyst.^36^ The water oxidation activity is very low for g-CN due to less conductivity and fast recombination of charge carriers.^37^ Notably, as compared to the observations for BiVO_4_ and g-CN, the water oxidation photocurrents are probably higher in the whole potential range for BiVO_4_/g-CN (6 mg). At 0.43 V, the water oxidation photocurrent densities are 0.03, 0.12 and 0.47 mA cm^−2^ for g-CN, BiVO_4_ and BiVO_4_/g-CN (6 mg), respectively. The photocurrent density of BiVO_4_/g-CN (6 mg) is ∼4 times higher than that for pristine BiVO_4_. The water oxidation onset potentials are −0.449 V and −0.41 V for BiVO_4_/g-CN (6 mg) and BiVO_4_, respectively (Fig. 8(a)). The slightly negative shift for BiVO_4_/g-CN (6 mg) may be caused by the compact contact of BiVO_4_ with g-CN, which is favorable for the fast transfer of the photogenerated holes from BiVO_4_ to the reaction interface. The improved charge carrier separation of BiVO_4_/g-CN (6 mg) can be further verified by electrochemical impedance spectroscopy (EIS) (Fig. 8(b)). The Nyquist plot of the BiVO_4_/g-CN (6 mg)-deposited electrode exhibits a smaller radius of the semicircle than that for individual g-CN and BiVO_4_-deposited electrodes, indicating less charge transfer resistance for BiVO_4_/g-CN (6 mg) than that for g-CN and BiVO_4_. In addition, the polarization curves of the photocatalyst samples with the potential from 0.38 to 1.2 V *versus* (Ag/AgCl) were measured (Fig. 8(c)). For BiVO_4_/g-CN (6 mg), the considerably high anodic current density indicated the boosted photo-response ability and photocatalytic activity than that of g-CN and BiVO_4_.

**Fig. 9:**
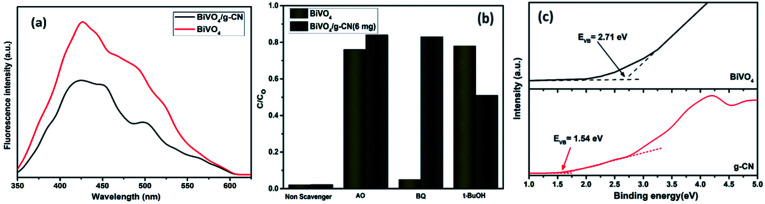
(a) PL spectral changes observed from BiVO_4_/g-CN and BiVO_4_ in a 5 × 10^−4^ M basic solution of terephthalic acid (excitation at 315 nm) irradiated by visible light; (b) trapping of active species during photocatalytic degradation experiment of MB over BiVO_4_/g-CN and BiVO_4_ under visible light irradiation after 40 min. (c) VB XPS spectra of g-CN and BiVO_4_.

Reactive oxygen species, especially hydroxyl radicals (·OH) and superoxide anion radicals (·O_2_^−^), have been considered to be the key species in the photocatalytic decomposition of many hazardous organic compounds due to their high reaction activity. PL of a photocatalyst has been defined as the measure of the charge separation efficiency. The charge separation efficiency greatly increased in the BiVO_4_/g-CN samples, which was proved by the decreased PL spectrum intensity, as shown in Fig. 9(a). This increased charge separation efficiency plays a key role in improving the photocatalytic activities because both g-CN and BiVO_4_ suffer from the fast recombination of electron–hole pairs, which severely limits their photocatalytic activities. Typically, three scavengers (*t*-BuOH, BQ and AO) were used to estimate the hydroxyl radicals (·OH), superoxide radicals (·O_2_^−^) and holes (h^+^), respectively.^38,39^ The above-mentioned three scavengers (1 mmol) were added to the reaction system along with BiVO_4_/g-CN (6 mg) or pure BiVO_4_.

As displayed in Fig. 9(b), when AO is added into the reaction system as an h^+^ scavenger along with BiVO_4_/g-CN (6 mg) or pure BiVO_4_, the photodegradation process was inhibited significantly, indicating that h^+^ can dominantly affects the decomposition of MB. The rate of MB removal was also significantly suppressed with the addition of the BQ scavenger for BiVO_4_/g-CN (6 mg) and on the other hand, it was not suppressed for pure BiVO_4_. This result demonstrated that the ·O_2_^−^ radical species significantly played a crucial role towards the degradation of MB by BiVO_4_/g-CN (6 mg). In addition, when the *t*-BuOH scavenger was added to capture ·OH species, the MB removal efficiency was reduced to some extent for BiVO_4_/g-CN (6 mg) but significantly suppressed for pure BiVO_4_, implying that to some extent, ·OH was also produced and participated in the photodegradation process by BiVO_4_/g-CN (6 mg).

**Fig. 10:**
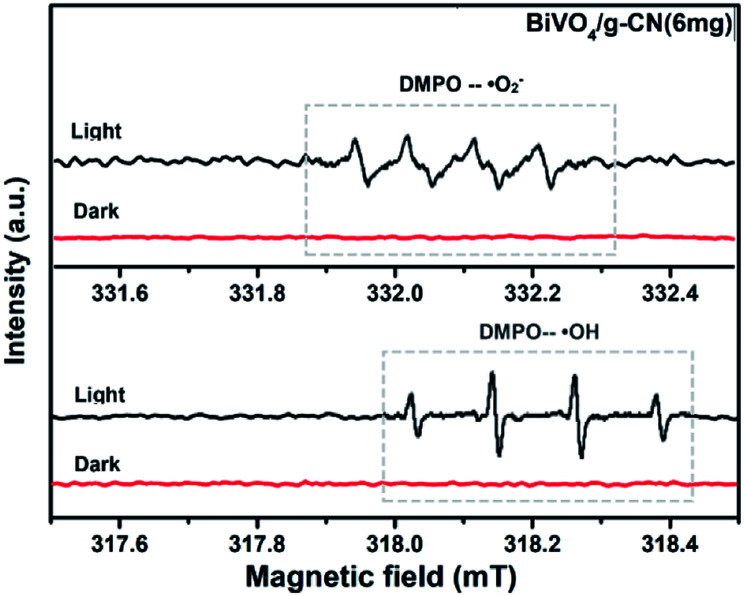
ESR spectrum of BiVO_4_/g-CN samples under irradiation: (a) in methanol dispersion for DMPO–˙O_2_^−^ and (b) in aqueous dispersion for DMPO–˙OH.

ESR is usually used to explore the reactive oxygen species evolved during the photocatalytic process. Fig. 10 shows the ESR spin-trap signals (with DMPO) of the samples in two different dispersions. From Fig. 10(a), the characteristic peaks of the DMPO–˙O_2_^−^ adducts are found for the visible-light-irradiated BiVO_4_/g-CN (6 mg) suspension in methanol. As shown in Fig. 10(b), four separate characteristic peaks with intensity 1 : 2 : 2 : 1 for the DMPO–˙OH adduct are observed for the suspension of BiVO_4_/g-CN (6 mg) in water under visible-light illumination, showing that the ·OH radicals are produced efficiently. However, in the dark and under identical conditions, no such signals were detected.

On the basis of all the above-mentioned results, we can preliminarily conclude that ·O_2_^−^ and h^+^ are the leading species in this photocatalytic reaction system for the BiVO_4_/g-CN (6 mg) heterojunction system. Meanwhile, the ·OH species also plays a certain role. It is commonly accepted that the overall photocatalytic performance is dependent on several factors, such as the specific surface area, photogenerated electron–hole transportation rate and photoresponse ability. The evaluation of the photocatalytic activity indicated that the composite with the optimal amount of g-CN (6 mg) showed remarkably enhanced performance due to the enlarged surface area (25 m^2^ g^−1^).

**Fig. 11:**
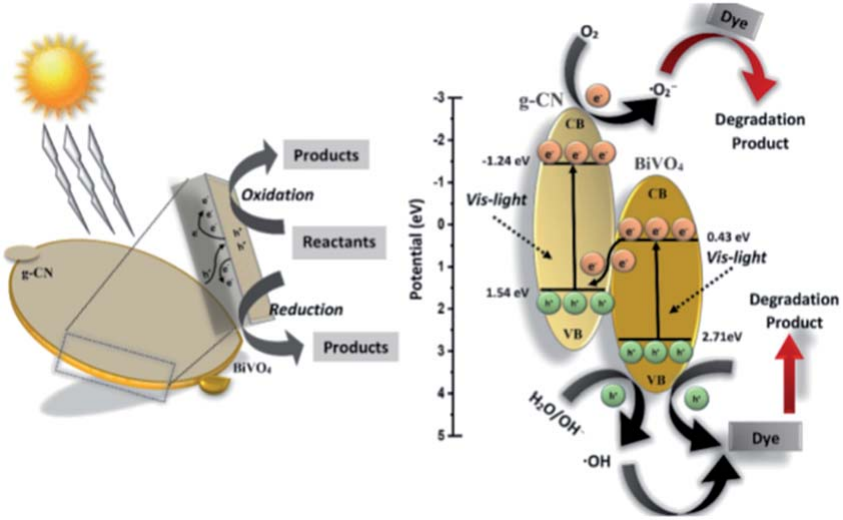
Schematic illustration of the proposed reaction mechanism in the BiVO_4_/g-CN (6 mg) nanocomposite-based reaction system towards dye (MB, BPB) degradation under visible light irradiation.

Thus, based on the afore-mentioned results, a proposed pathway mechanism for the photocatalytic reactions occurring on BiVO_4_/g-CN (6 mg) is demonstrated in Fig. 11. Fig. 9(c) demonstrates that the two constituent semiconductors show a well-staggered band alignment. The positions of the valence band (VB) and conduction band (CB) were calculated by the following empirical equation: *E*_VB_ = *E*_CB_ + *E*_g_; here, *E*_CB_ corresponds to the CB edge potential, *E*_VB_ corresponds to the VB edge potential and *E*_g_ represents the band gap of the samples. The VB and CB of the as-prepared m-BiVO_4_ were calculated to be +2.71 eV and +0.43 eV, while those of g-CN were +1.54 eV and −1.24 eV, respectively. The VB of g-CN was not much +ve than that of ·OH\OH^−^ (2.4 eV) and the CB of m-BiVO_4_ was not much −ve than that of ·O_2_^−^\O_2_ (−0.33 eV);^40^ this means that the ·OH and ·O_2_^−^ species cannot be generated based on the traditional heterojunction-transfer process. However, it was demonstrated that the ·OH and ·O_2_^−^ species were generated and participated in this photocatalytic degradation reaction system. Therefore, the *Z*-scheme mechanism was more suitable to describe the photocatalytic reaction process. In other words, the photogenerated e^−^ generated on BiVO_4_ (CB) prefers to transfer to g-CN (VB) and recombine with the remaining holes. Then, the e^−^ in g-CN (CB) reacts with the dissolved O_2_ to generate ·O_2_^−^ for dye (MB) degradation. The holes in BiVO_4_ (VB) would participate in the photocatalytic reaction process *via* dual pathways: on one hand, the holes can react directly with MB; on the other hand, the holes can also react with water (H_2_O) to generate ·OH and then as a degradation product degrade MB. Meanwhile, due to the lack of the transfer process, the remaining holes and electrons in the VB of BiVO_4_ and the CB of g-CN influenced strong redox ability, which is an important factor to enhance the photocatalytic performance based on the prepared BiVO_4_ and g-CN hybrid composites. Furthermore, the intrinsic material properties, *i.e.*, the effective surface coverage of the g-CN nanosheets on the m-BiVO_4_ networks leads to more reactive sites by completely exposing the adjacent area of layer interfaces to the solution. Similarly, the separation of electron–hole pairs is facilitated by the small size of the g-CN nanosheets with a decreased charge transport distance.^41^

## 4 Conclusion

A novel BiVO_4_ and graphitic carbon nitride-grafted (BiVO_4_/g-CN) photocatalytic system is developed with superior visible light harvesting ability and efficient photocatalytic activity both for organic degradation and water oxidation applications. The boosted photocatalytic activity and removal efficiency can be attributed to the synergistic effects of g-CN and BiVO_4_. The enhanced light response ability in the whole spectrum and the larger specific surface area of g-CN in the reaction process can provide a large amount of reaction sites and lead to high photocatalytic performance. The intimate connects and the matching of the band gap can promote the transfer and separation of the photogenerated electrons and holes. A possible *Z*-scheme mechanism of the BiVO_4_/g-CN composite from this synthetic system was proposed based on the PL and trapping tests. The h^+^ and ·O_2_^−^ were the main active species, with an order of h^+^ > ·O_2_^−^, for the MB and BPB degradation. Moreover, the excellent stability and recycling ability of our BiVO_4_/g-CN (6 mg) photocatalyst ensure its practical applications in environmental remediation.

## Conflicts of interest

There are no conflicts to declare.

## Supplementary Material

RA-010-C9RA09473C-s001

## References

[cit1] Khin M. M., Nair A. S., Babu V. J., Murugan R., Ramakrishna S. (2012). Energy Environ. Sci..

[cit2] Hong X., Kim J., Shi S.-F., Zhang Y., Jin C., Sun Y., Tongay S., Wu J., Zhang Y., Wang F. (2014). Nat. Nanotechnol..

[cit3] Jiang Z., Qian K., Zhu C., Sun H., Wan W., Xie J., Li H., Wong P. K., Yuan S. (2017). Appl. Catal., B.

[cit4] Kuriki R., Matsunaga H., Nakashima T., Wada K., Yamakata A., Ishitani O., Maeda K. (2016). J. Am. Chem. Soc..

[cit5] Sun S., Shen G., Jiang J., Mi W., Liu X., Pan L., Zhang X., Zou J. J. (2019). Adv. Energy Mater..

[cit6] Zhang Y. C., Afzal N., Pan L., Zhang X., Zou J. J. (2019). Adv. Sci..

[cit7] Wu W., Li X., Ruan Z., Li Y., Xu X., Yuan Y., Lin K. (2018). Inorg. Chem. Front..

[cit8] Chen X., Zhang J., Fu X., Antonietti M., Wang X. (2009). J. Am. Chem. Soc..

[cit9] Wen J., Xie J., Zhang H., Zhang A., Liu Y., Chen X., Li X. (2017). ACS Appl. Mater. Interfaces.

[cit10] She X., Wu J., Xu H., Zhong J., Wang Y., Song Y., Nie K., Liu Y., Yang Y., Rodrigues M. T. F. (2017). Adv. Energy Mater..

[cit11] Li M., Zhang L., Wu M., Du Y., Fan X., Wang M., Zhang L., Kong Q., Shi J. (2016). Nano Energy.

[cit12] Lin Z., Wang X. (2013). Angew. Chem., Int. Ed..

[cit13] Ahmed T., Zhang H.-l., Gao Y.-Y., Xu H.-b., Zhang Y. (2018). Mater. Res. Bull..

[cit14] Liu H., Hou H., Gao F., Yao X., Yang W. (2016). ACS Appl. Mater. Interfaces.

[cit15] Hu J., Chen W., Zhao X., Su H., Chen Z. (2018). ACS Appl. Mater. Interfaces.

[cit16] Wang M., Liu Q., Che Y., Zhang L., Zhang D. (2013). J. Alloys Compd..

[cit17] Wang Y., Wang W., Mao H., Lu Y., Lu J., Huang J., Ye Z., Lu B. (2014). ACS Appl. Mater. Interfaces.

[cit18] Zhao W., Wang Y., Yang Y., Tang J., Yang Y. (2012). Appl. Catal., B.

[cit19] Zhou P., Yu J., Jaroniec M. (2014). Adv. Mater..

[cit20] Li K., Zhang R., Gao R., Shen G.-Q., Pan L., Yao Y., Yu K., Zhang X., Zou J.-J. (2019). Appl. Catal., B.

[cit21] Xu H., Yi J., She X., Liu Q., Song L., Chen S., Yang Y., Song Y., Vajtai R., Lou J. (2018). Appl. Catal., B.

[cit22] Huang Z.-F., Song J., Wang X., Pan L., Li K., Zhang X., Wang L., Zou J.-J. (2017). Nano Energy.

[cit23] Jia X., Tahir M., Pan L., Huang Z.-F., Zhang X., Wang L., Zou J.-J. (2016). Appl. Catal., B.

[cit24] Li C., Wang S., Wang T., Wei Y., Zhang P., Gong J. (2014). Small.

[cit25] Lee S. C., Lintang H. O., Yuliati L. (2012). Chem.–Asian J..

[cit26] Ahmed T., Zhang H.-l., Xu H.-b., Zhang Y. (2017). Colloids Surf., A.

[cit27] Xu Z., Zhuang C., Zou Z., Wang J., Xu X., Peng T. (2017). Nano Res..

[cit28] Ho W., Zhang Z., Xu M., Zhang X., Wang X., Huang Y. (2015). Appl. Catal., B.

[cit29] He Y., Zhang L., Teng B., Fan M. (2014). Environ. Sci. Technol..

[cit30] Tian N., Huang H., He Y., Guo Y., Zhang T., Zhang Y. (2015). Dalton Trans..

[cit31] She X., Xu H., Xu Y., Yan J., Xia J., Xu L., Song Y., Jiang Y., Zhang Q., Li H. (2014). J. Mater. Chem. A.

[cit32] Ran J., Ma T. Y., Gao G., Du X.-W., Qiao S. Z. (2015). Energy Environ. Sci..

[cit33] Xia P., Zhu B., Cheng B., Yu J., Xu J. (2017). ACS Sustainable Chem. Eng..

[cit34] Giménez S. and Bisquert J., Photoelectrochemical solar fuel production, Springer International Publishing, 2016, ch. 8, pp. 361–362

[cit35] Gawande S. B., Thakare S. R. (2012). Int. Nano Lett..

[cit36] Zhang Y., Mori T., Niu L., Ye J. (2011). Energy Environ. Sci..

[cit37] Liu C., Zhang Y., Dong F., Reshak A., Ye L., Pinna N., Zeng C., Zhang T., Huang H. (2017). Appl. Catal., B.

[cit38] Ng C., Iwase A., Ng Y. H., Amal R. (2012). J. Phys. Chem. Lett..

[cit39] Li K., Zeng X., Gao S., Ma L., Wang Q., Xu H., Wang Z., Huang B., Dai Y., Lu J. (2016). Nano Res..

[cit40] Deng Y., Tang L., Zeng G., Wang J., Zhou Y., Wang J., Tang J., Liu Y., Peng B., Chen F. (2016). J. Mol. Catal. A: Chem..

[cit41] Auerbach E. A., Seyfried E. E., McMahon K. D. (2007). Water Res..

