# Supplementary material for: Z-scheme 2D-m-BiVO4 networks decorated by a g-CN nanosheet heterostructured photocatalyst with an excellent response to visible light
Source: RSC Adv. 2020 Jan 20;10(6):3192–202. doi: 10.1039/c9ra09473c (PMC9048574; doi:10.1039/c9ra09473c)
Supplement: RA-010-C9RA09473C-s001 [file RA-010-C9RA09473C-s001.pdf]

Electronic Supplementary Material (ESI) for RSC Advances.
This journal is © The Royal Society of Chemistry 2019

Supplementary Information

Z-scheme 2D-m-$BiVO_4$ Networks Decorated by g-CN Nanosheets Heterostructured Photocatalyst with Excellent Response to Visible Light

Toheed Ahmed[a,b,c*], Muhammad Ammar[b,d*], Aimen Saleem[e], Hong-ling Zhang[a], Hong-bin Xu[a]

[a] Key Laboratory of Green Process and Engineering, Institute of Process Engineering, Chinese Academy of Sciences, Beijing 100190, China.

[b] University of Chinese Academy of Sciences, Beijing 100049, China.

[c] Department of Applied Chemistry, Government College University, Faisalabad 38000, Pakistan.

[d] Department of Chemical Engineering Technology, Government College University, Faisalabad 38000, Pakistan.

[e] Biotechnology and Fermentation Group, Department of Animal Sciences, The Ohio State University and QARDC, Wooster Ohio 44691, USA.

*** Corresponding author:** toheedahmed@gcuf.edu.pk (T. Ahmed), mammar@gcuf.edu.pk (M. Ammar), Tel/Fax: +92419203027.

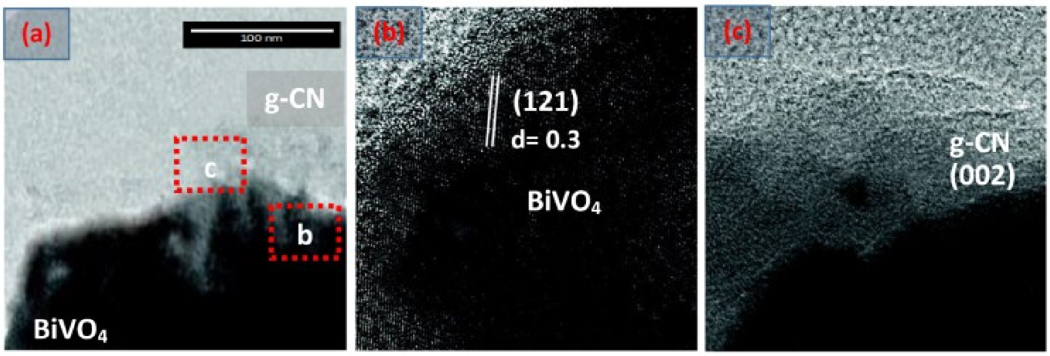

Fig. S1 TEM images of the as-obtained samples: (a) BiVO4, (c) $BiVO_4$/g-CN (6mg); HRTEM images of (b, c) $BiVO_4$/g-CN (6mg).

.

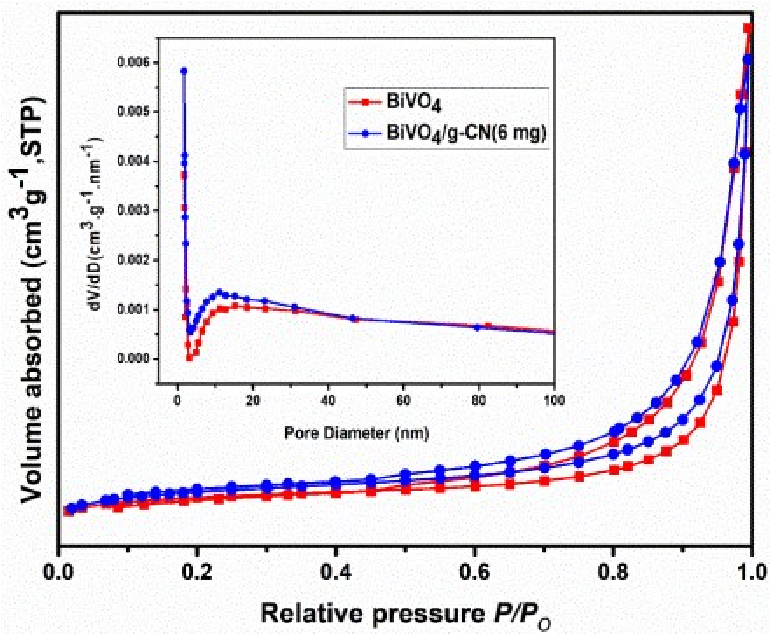

Fig. S2 Nitrogen adsorption/desorption isotherms, and pore-size distributions of the as-prepared samples.

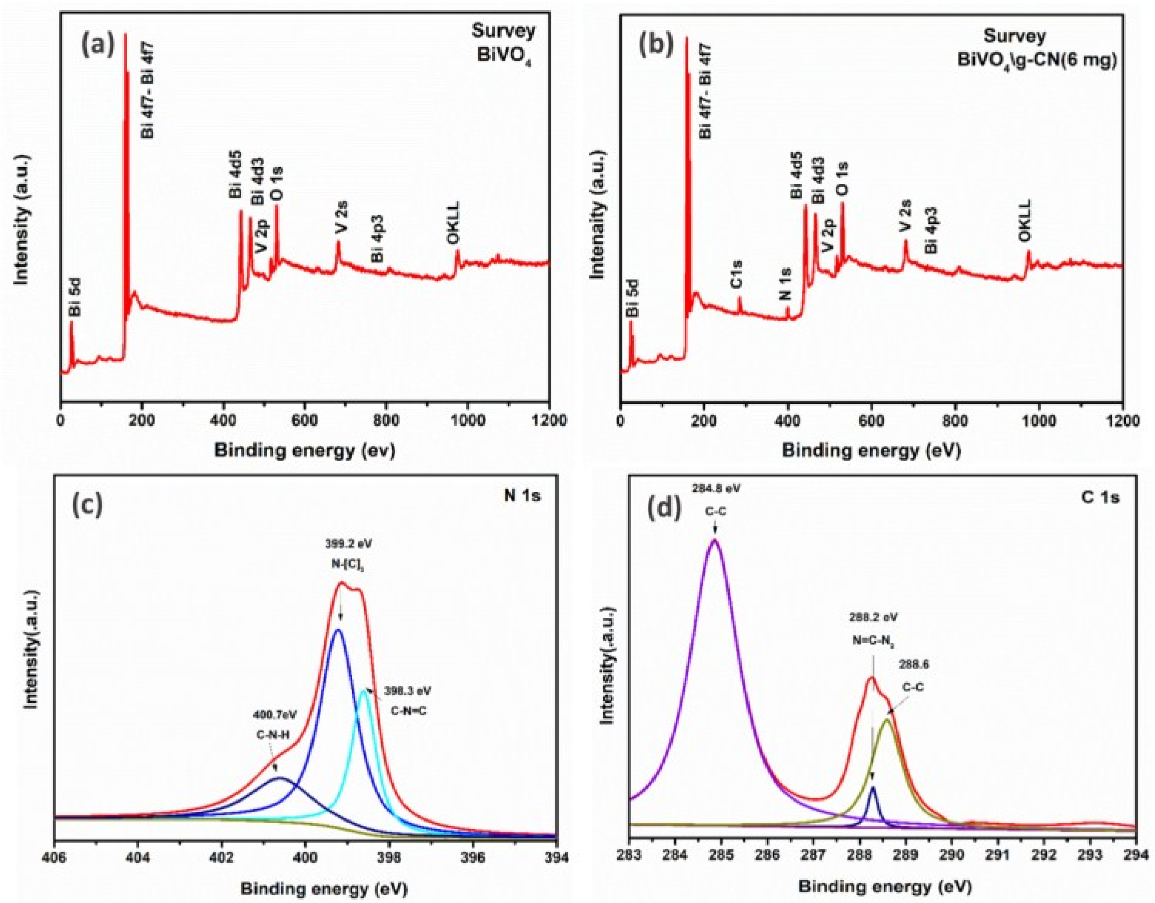

Fig. S3 XPS survey spectrum of (a) $BiVO_4$ (b) $BiVO_4$/g-CN (6mg), XPS spectra of $BiVO_4$/g-CN (6mg) (c) N1s (d) C1s.

Table S1 Surface properties of prepared pure $BiVO_4$ and $BiVO_4$/g-CN (6 mg).

**Samples**	**Pore Volume (cm^3g^{-1})**	**Average Particle Size (nm)**	**Average Pore Size (nm)**
$BiVO_4$	0.15133	239.6738	24.7082
$BiVO_4$/g-CN (6 mg)	0.12529	290.5873	31.1876
